# Surgical Techniques for Tricuspid Valve Disease

**DOI:** 10.3389/fcvm.2018.00118

**Published:** 2018-08-28

**Authors:** Igor Belluschi, Benedetto Del Forno, Elisabetta Lapenna, Teodora Nisi, Giuseppe Iaci, David Ferrara, Alessandro Castiglioni, Ottavio Alfieri, Michele De Bonis

**Affiliations:** Department of Cardiac Surgery, IRCCS San Raffaele University Hospital, Milan, Italy

**Keywords:** tricuspid, valve, surgery, techniques, disease

## Abstract

Tricuspid valve disease affects millions of patients worldwide. It has always been considered less relevant than the left-side valves of the heart, but this “forgotten valve” still represents a great challenge for the cardiac surgeons, especially in the most difficult symptomatic scenarios. In this review we analyze the wide spectrum of surgical techniques for the treatment of a diseased tricuspid valve.

## Introduction

The disease of TV generally appears in the form of regurgitation. Secondary or functional disease represents the more suitable case for a repair, while the organic or primary deterioration of the valve is unlikely to be repaired. Over the past four decades, many procedures have been described, varying from simple sutures to a large number of prosthetic rings. The present evidence reports a superiority of the repair over replacement, in particular when annuloplasty is associated. Here we summarize the most commonly used techniques to repair this forgotten valve or replace it in the worst cases.

## Tricuspid valve repair techniques

### Functional disease

When pulmonary hypertension develops as a consequence of a left-heart pathology, a functional tricuspid regurgitation may appear. In this case the valve is not affected by organic lesions, nevertheless an annular dilatation may be associated.

Current guidelines suggest treating this disease when the regurgitation becomes severe and only if a left-heart surgery is planned. If the grade of insufficiency is less than severe, surgery should be performed in case of a septal-anterior diameter ≥ 40 mm (or ≥21 mm/m^2^). If there are signs of progressive right ventricle dysfunction or dilatation (in the absence of left or right ventricle severe decline or pulmonary hypertension), even if in asymptomatic patients, a reintervention for tricuspid regurgitation is needed after a previous left-side surgery (1).

The key of the tricuspid valve repair consists of the reduction in the right ventricle after-load and in the annulus diameter (2).

#### Suture annuloplasty

##### Kay procedure

**Figure 1:**
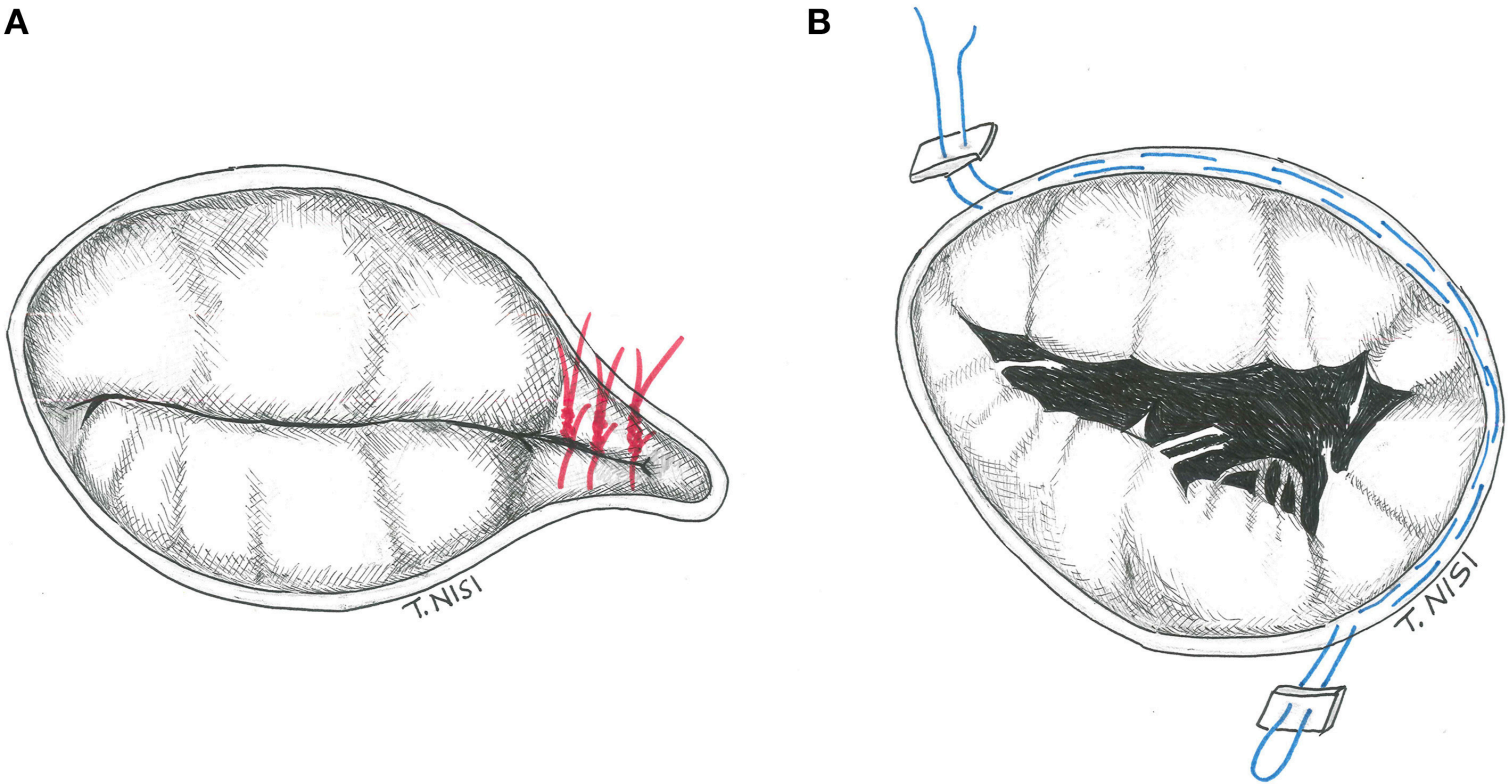
**(A)** The Kay procedure. **(B)** The De Vega technique. [With permission of De Bonis et al. (3)].

In 1965 Kay et al. described, for the first time, a repair technique to treat secondary tricuspid regurgitation. Using a 1-0 silk suture (placed through the posterior leaflet and the commissures), the posterior leaflet is completely excluded, and a functional bicuspid valve is finally obtained. It is preferable to put other sutures to reinforce the first stitch. In addition, some variants (i.e., the positioning of some pledgets) could be performed (Figure 1A).

##### De vega procedure

The De Vega annuloplasty has been proposed in 1972. This procedure consists of reducing the area of the tricuspid annulus and rapidly became the most popular technique for the treatment of annular dilatation. It is generally performed by two 2-0 Ti-cron or 4-0 polypropylene running parallel sutures (with 5–6 mm bites), starting on the postero-septal commissure, through the endocardium, and directed around the perimeter of the orifice in a counterclockwise direction reaching the antero-septal commissure. The other parallel suture is placed about 1–2 mm outside the previous one, and finally tied together (Figure 1B) (4). In case of fragile endothelium, the sutures could cut the annulus, therefore some pledgets may be positioned between every bite to reinforce the annuloplasty as proposed by Antunes and Girdwood in 1983 (5).

#### Ring annuloplasty

**Figure 2:**
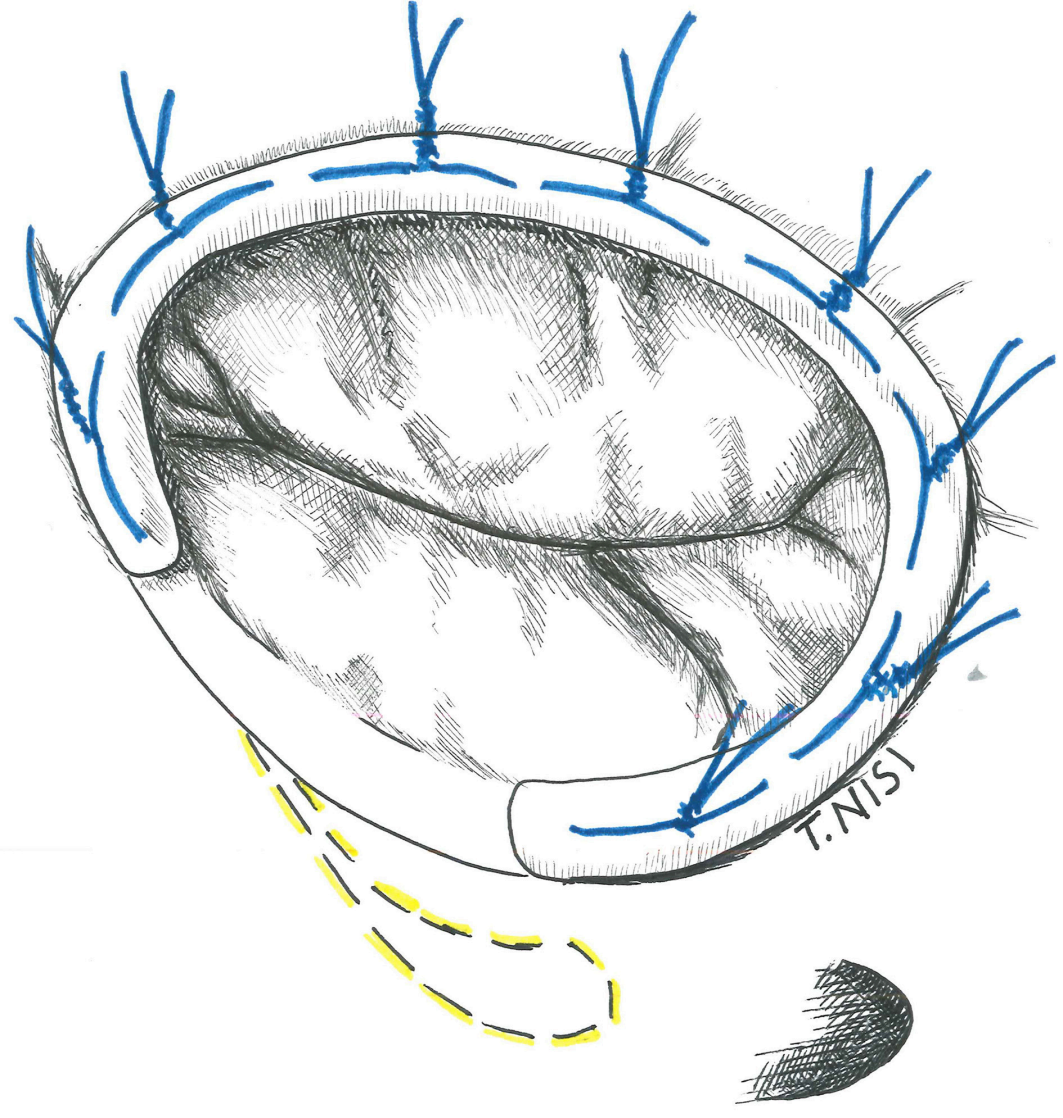
The image shows a tricuspid ring annuloplasty. [With permission of De Bonis et al. (3)].

The idea of a prosthetic ring to reinforce the tricuspid annulus was first introduced by Carpentier in 1971. Rigid or semi-rigid ring has been designed to fix the annulus during systole, restoring the physiologic geometry of the valve, while flexible ones may be used as well to reduce the annular dilatation, but failed to restore the 3D morphology (6, 7). The right size of the ring is chosen by measuring the distance from the antero-septal to postero-septal commissures (i.e., the surface of the anterior leaflet) and the ring is then implanted using eight to ten 2-0 Ti-cron stitches starting posteriorly (at the midpoint of the septal leaflet) and then proceeding counterclockwise. The surgeon must pay attention during the placement of stitches to damage the conduction system and to avoid the aortic root at the level of septal and anterior leaflet, respectively. The last stitch is placed above the antero-septal commissure, and the ring is finally parachuted and fixed (Figure 2).

#### Enlargement of the anterior leaflet

Sometimes, an isolated annuloplasty is not sufficient to correct the disease, especially in case of severe tethering. As a consequence, in 2008 Dreyfus et al. proposed the enlargement of the anterior leaflet to solve this problem. After removing the native anterior leaflet, a piece of autologous pericardium is prepared by measuring the length as the distance between the antero-septal and the antero-posterior commissures, whereas the height as the greatest distance between the detached leaflet and the annulus. Finally, the patch is sutured with a polypropylene 5-0 suture to the annulus and a semi-rigid annuloplasty ring is implanted (8).

#### “Clover technique”

**Figure 3:**
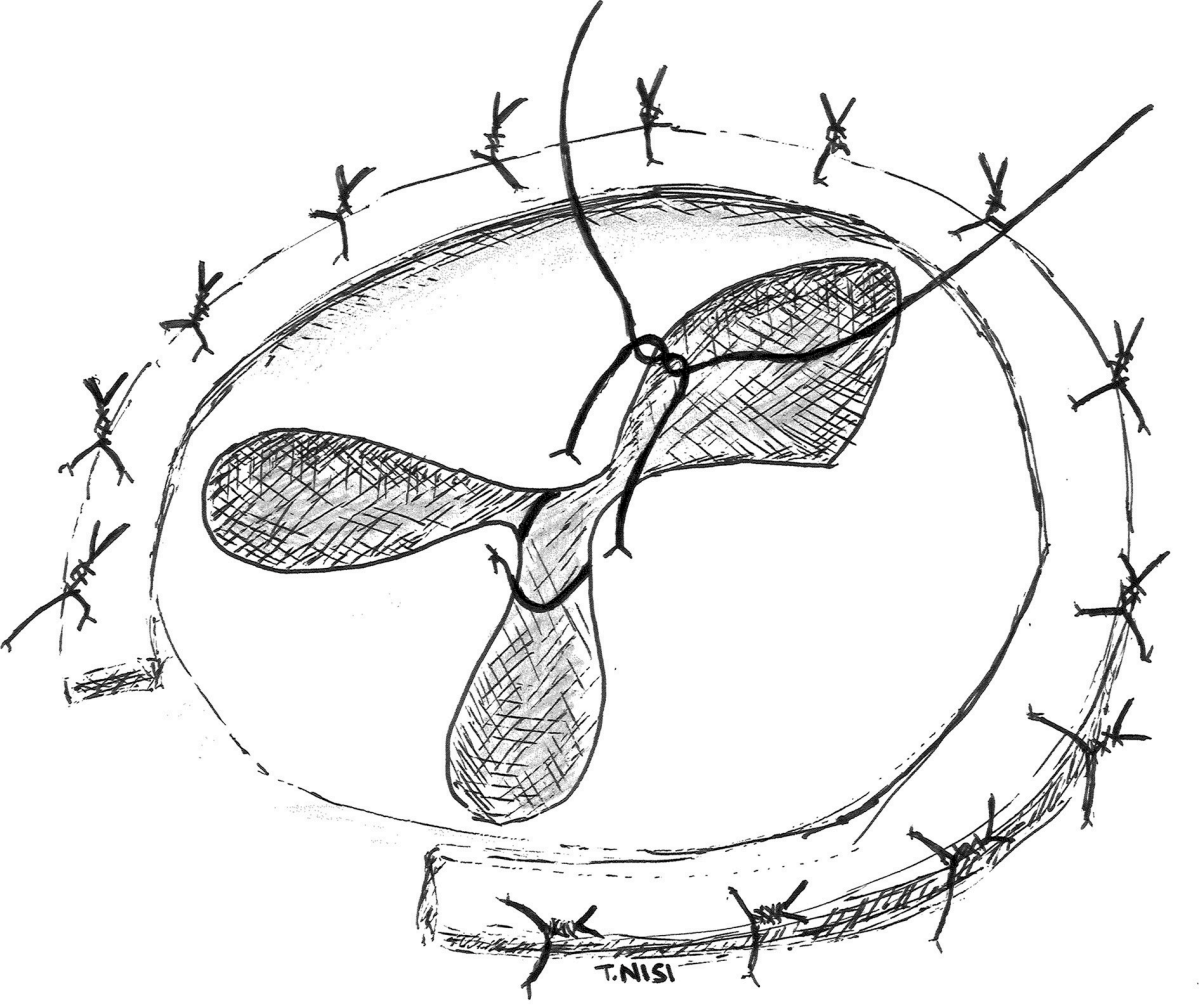
In this picture the “Clover Technique” by Alfieri et al. is reported. The leaflets are joined together, and the valve finally assumes a shape like a clover. [With permission of De Bonis et al. (3)].

In 2003 Alfieri et al. presented a technique for the correction of severe functional tricuspid insufficiency in case of important tethering. It consists of stitching together the middle point of the free edges of the tricuspid leaflets by using a 5.0 polipropylene suture without pledgets and adding a semi-rigid ring. At this point, the valve became clover-shaped, so this technique has been called “the Clover Technique.” It was first introduced for the treatment of post-traumatic tricuspid regurgitation, while later became effective even in complex cases both of primary or secondary tricuspid regurgitation (9) (Figure 3).

#### Results

The failure rate for the treatment of tricuspid valve by using a suture or a ring annuloplasty at one month after surgery ranges from 8 to 15% (10, 11). Risk factors include: the severity of preoperative tricuspid regurgitation, presence of pacemakers, pulmonary hypertension, LV dysfunction, increased left ventricular remodeling, severe tethering of the tricuspid leaflets and the use of suture rather than ring annuloplasty. Nevertheless, many observational studies and RCTs compared the two types of annuloplasty (suture or ring): the conclusion was that the placement of a ring is associated with a more durable repair, especially when severe tricuspid annular dilation or pulmonary hypertension are present (12, 13). In addition, when compared to suture annuloplasty, tricuspid rings also provide better long-term and event-free survival up to 15 years after surgery (14, 15). In case of severe tethering associated to annular dilatation, annuloplasty alone is unlikely to be durable (16) so an additional procedure, such as the enlargement of the anterior leaflet or “clover technique,” may be used to obtain a more durable repair (8, 9). However, more studies are mandatory to prove the long-term outcome of these procedures.

### Tricuspid valve repair for primary disease

The organic disease of the tricuspid valve (i.e., primary insufficiency) is most commonly caused by degenerative valve disease or bacterial endocarditis (generally in western countries), while rheumatic disease still remains the most prevalent form in developing countries.

Leaflet lesions may include: excess of tissue, thickening, perforation and tear; whereas the chordae tendinae and the papillary muscles may be fused (especially in the rheumatic disease), elongated or damaged. Restoring the normal leaflet mobility, ensuring an adequate surface of coaptation, is the aim of the techniques reported, always followed by ring annuloplasty.

#### Intervention on the leaflets

**Figure 4:**
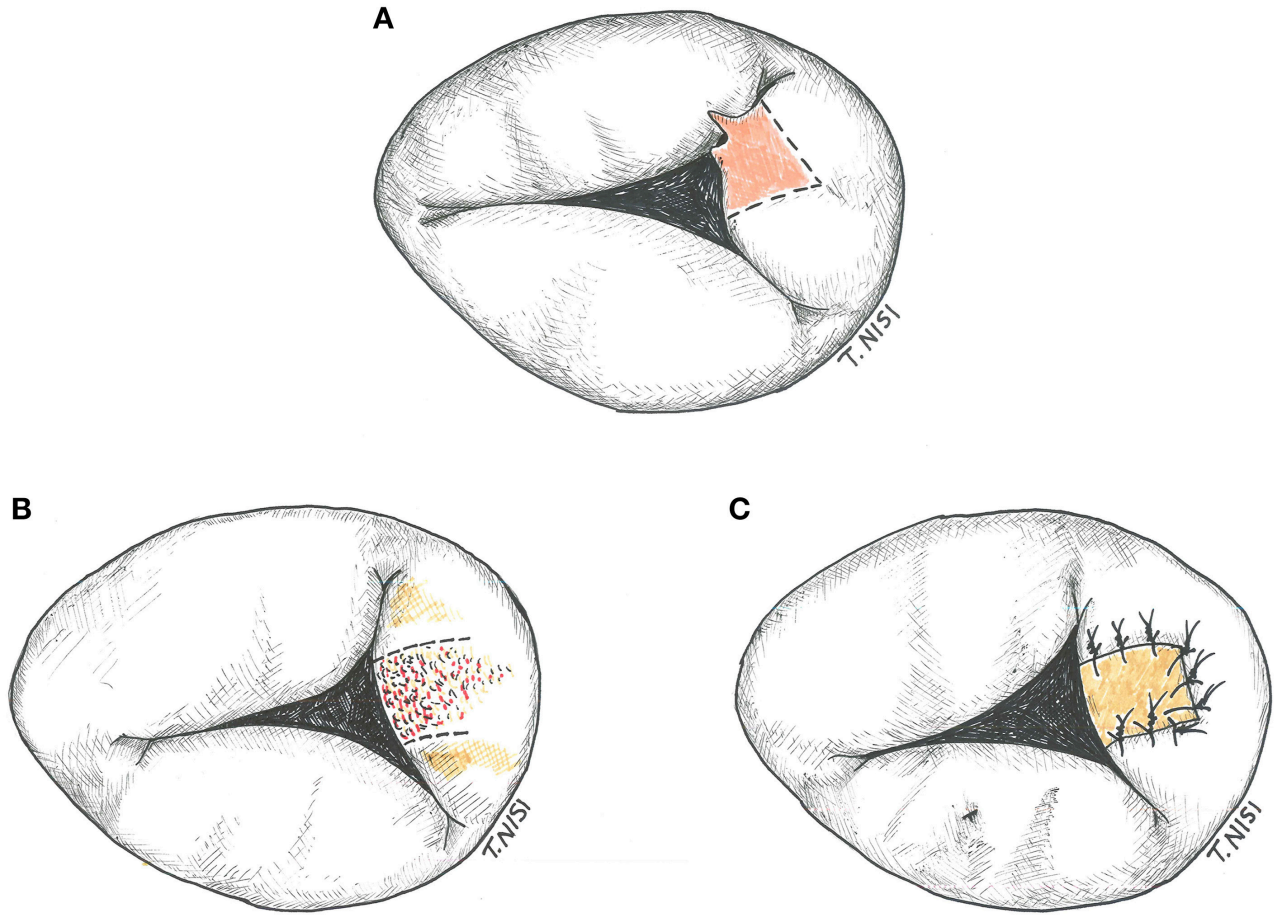
**(A)** Tricuspid triangular resection. **(B)** Resection of the leaflet in a case of bacterial endocarditis. **(C)** The gap is restored by using an autologous pericardium patch. [With permission of De Bonis et al. (3)].

If the prolapsing segment is less than one tenth of the leaflet surface area and the chordal rupture is small, a triangular resection can be performed. After resection, a 5-0 polypropylene running suture or interrupted stitches are used for the leaflet synthesis. An annular plication and/or a pericardial patch may be used in case of endocarditis (Figure 4).

#### Intervention on the chordae

In case of important chordal rupture both chordal transposition and artificial chordae implantation may be adopted, as for the mitral valve repair. In the first case, a small segment of adjacent non-prolapsing leaflet is resected and then implanted on the prolapsing one using 5-0 polypropylene running suture.

Otherwise, for the implantation of artificial chordae, an accurate measurement of native chordae of a non-prolapsing leaflet is performed and used to choose the proper length. Then the neo-chordae are implanted on the corresponding papillary muscle and finally on the free margin of the prolapsing leaflet.

#### Intervention on the papillary muscles

A sliding papillary muscle plasty technique may be the best choice to correct a diseased papillary muscles or extensive chordal elongation. The elongated papillary muscle, or the papillary muscle underlying the elongated chordae, is lowered to the proper level and fixed to the adjacent one using 5-0 polypropylene interrupted stitches.

#### Intervention on the commissures

When the rheumatic disease is present, it may provoke a fusion of the three commissures (generally associated with that of the underlying chordae). The surgical technique consists of performing commissurotomy and dividing fused chordae using an 11 blade under direct visualization.

## Tricuspid valve replacement

Sometimes the valve structure is dramatically compromised (such as in endocarditis, carcinoid syndrome, and radiation induced disorder), therefore reconstructive procedures may result not adequate and the decision for tricuspid valve replacement should be taken. Even in extreme dilatation due to functional tricuspid regurgitation, valve replacement should be considered. The choice of using a cardioplegic heart arrest or a beating heart procedure depends on the surgeon's preference and the risk of the patients, but sometimes combined techniques are used as well.

### Choice of the prosthetic valve

The choice of the prosthesis type (whether biological or a mechanical) should follow the same algorithm used for other cardiac valves (i.e., age, pathology, comorbidities etc.). By the way, the surgeon must remember that the specific right-heart low-pressure chambers and lower level of prostacyclin (a powerful inhibitor of platelet aggregation) may increase the risk of valve thrombosis (17, 18).

Furthermore, the mechanical choice could be prohibitive for a future pacemaker need. Given that, biological prostheses would seem to be an ideal solution. In addition, reoperations rate after bio-prosthetic tricuspid valve replacement seems to be lower compared to bio-prosthetic mitral valve replacements, and this may be due to the lower pressure and to the limited life expectancy in TVR patients (19). On the other hand, other studies reported similar survival rates (20–22). Finally, mechanical valve thrombosis is rarely fatal and often is successfully treated increasing anticoagulation therapy or using thrombolysis (23). However, in case of small size—and/or OAT—patients, the mechanical choice could be preferred (24).

It is not possible to state that there is a “gold standard” for prosthetic selection in tricuspid valve replacement. As a consequence, this choice should be strictly tailored considering the patients' profile (i.e., young patients <40 y.o. may benefit a mechanical TVR).

### Surgical technique

**Figure 5:**
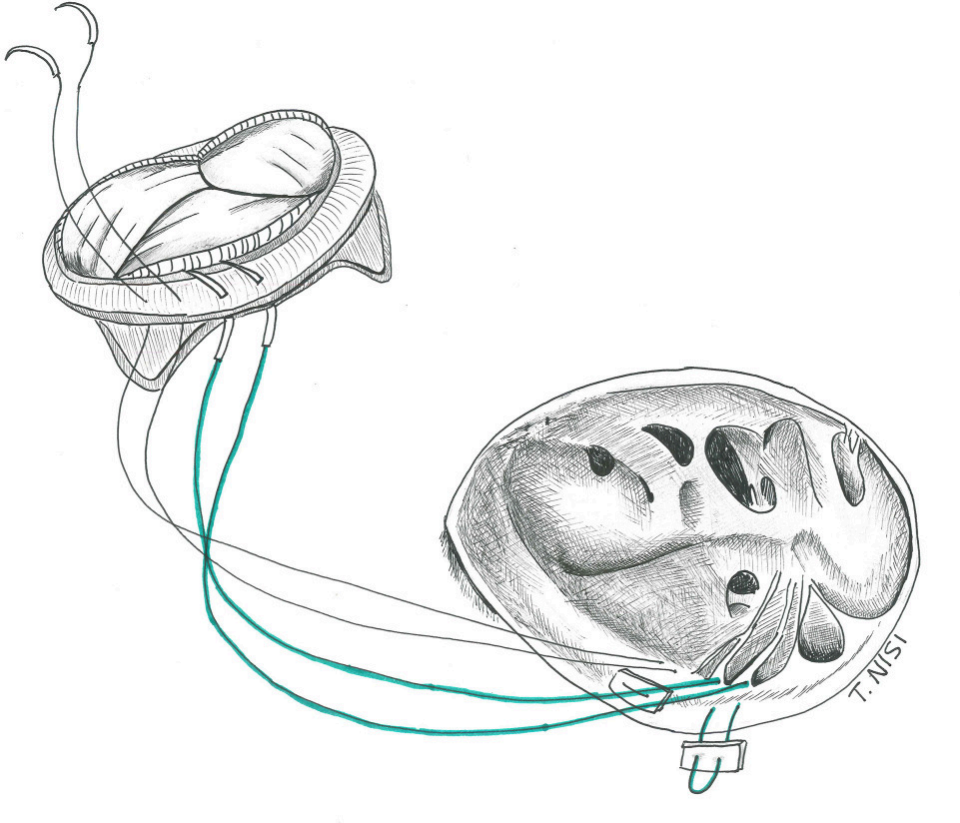
Tricuspid valve replacement using a bio-prosthesis. [With permission of De Bonis et al. (3)].

The native leaflets are generally resected (or fenestrated after the TVR in case of RVOT obstruction), leaving a 2 to 3 mm fringe of tissue on the annulus, dividing the chordal attachments deep in the right ventricle and keeping the septal leaflet *in situ*. Then the suture is performed using an everting 2-0 or 3-0 pledgeted Ti-cron stitch, along the circumference of the annulus from the atrial to the ventricular side of the valve, starting at the anterior leaflet and proceeding clockwise. Alternatively, when there is a need for a quicker procedure, pledgets sutures are used only at the level of the septal leaflet, while two continuous 2-0 Ti-cron sutures are used for the remaining circumference of the valve. An accurate sizing is crucial to avoid obstruction and septal lesions. The bio-prosthetic valve must be properly oriented to avoid obstruction of the right ventricle outflow tract considering its stent posts (which should stay at the 12, 4, and 8 o'clock positions). After passing the sutures through the sewing ring and slipping down the prosthesis, the sutures are finally secured starting from the septal leaflet (Figure 5).

### Results

The sub-optimal results of tricuspid valve replacement have always represented a difficult argument in cardiac surgery. As reported in many series, the replacement of tricuspid valve is generally adopted in reoperations, which represents itself an adjunctive intrinsic operative risk (20, 25). Some studies showed an operative mortality of 18%. NYHA class, female gender, bilirubin level, preoperative diuretic dose and preoperative hemoglobin level are all associated with increased operative mortality (25, 26). Late referral is the main issue in the management of these patients: the presence of enlarged and dysfunctioning right ventricles severely conditioned the operative results. The choice of a beating-heart technique without the need of aortic cross-clamping, generally in redo cases, represents an optimal tool and has showed acceptable acute mortality, especially if performed in the absence of ascites, significant right ventricular dysfunction and pulmonary hypertension (27). Regarding the outcomes on long-term, they are acceptable considering the clinical conditions of these patients. However, the presence of risk factors such as pulmonary hypertension, age at intervention and redo surgery, seem to have an impact on survival at follow- up (26, 27).

## Conclusion

This review provides an overview of the surgical techniques for the treatment of functional and organic tricuspid regurgitation. When possible, valve repair still remains the most useful procedure, while replacement is generally preferred in the most demanding cases. Surgeons must know the wide spectrum of this surgical techniques. Only the accurate choice of the most appropriate procedure will provide optimal and long-term results.

## Author contributions

BD and IB: equally draft the manuscript; TN: drawn the anatomical pictures; GI and DF: reviewed the manuscript; EL, AC, OA, and MD: supervised the manuscript.

### Conflict of interest statement

The authors declare that the research was conducted in the absence of any commercial or financial relationships that could be construed as a potential conflict of interest.
